# A20 plays a critical role in the immunoregulatory function of mesenchymal stem cells

**DOI:** 10.1111/jcmm.12849

**Published:** 2016-03-29

**Authors:** Rui‐Jie Dang, Yan‐Mei Yang, Lei Zhang, Dian‐Chao Cui, Bangxing Hong, Ping Li, Qiuxia Lin, Yan Wang, Qi‐Yu Wang, Fengjun Xiao, Ning Mao, Changyong Wang, Xiao‐Xia Jiang, Ning Wen

**Affiliations:** ^1^ Department of StomatologyChinese PLA General HospitalHaidian DistrictBeijingChina; ^2^ Department of Advanced Interdisciplinary StudiesInstitute of Basic Medical SciencesHaidian DistrictBeijingChina; ^3^ Department of Biology and Chemical EngineeringTongren UniversityTongren CityGuizhouChina; ^4^ Department of AnesthesiologyBeijing Aiyuhua Hospital for Children and WomenBeijingChina; ^5^ Center for Cell and Gene TherapyBaylor College of MedicineHoustonTXUSA; ^6^ Department of Experimental HematologyInstitute of Radiation MedicineBeijingChina

**Keywords:** mesenchymal stem cells, A20, immunoregulation

## Abstract

Mesenchymal stem cells (MSCs) possess an immunoregulatory capacity and are a therapeutic target for many inflammation‐related diseases. However, the detailed mechanisms of MSC‐mediated immunosuppression remain unclear. In this study, we provide new information to partly explain the molecular mechanisms of immunoregulation by MSCs. Specifically, we found that A20 expression was induced in MSCs by inflammatory cytokines. Knockdown of A20 in MSCs resulted in increased proliferation and reduced adipogenesis, and partly reversed the suppressive effect of MSCs on T cell proliferation *in vitro* and inhibited tumour growth *in vivo*. Mechanistic studies indicated that knockdown of A20 in MSCs inhibited activation of the p38 mitogen‐activated protein kinase (MAPK) pathway, which potently promoted the production of tumour necrosis factor (TNF)‐α and inhibited the production of interleukin (IL)‐10. Collectively, these data reveal a crucial role of A20 in regulating the immunomodulatory activities of MSCs by controlling the expression of TNF‐α and IL‐10 in an inflammatory environment. These findings provide novel insights into the pathogenesis of various inflammatory‐associated diseases, and are a new reference for the future development of treatments for such afflictions.

## Introduction

Mesenchymal stem cells (MSCs) can be found in nearly all tissues, including muscle, adipose, umbilical cord, dental pulp and bone marrow 1, 2, 3. Mesenchymal stem cells are defined as self‐renewing multi‐potent non‐haematopoietic progenitor cells capable of differentiating into tissue‐specific cell types, including those of bone, cartilage, muscle, adipose and other tissue types 4, 5.

Many studies have found that MSCs possess immunoregulatory capabilities and are thus a therapeutic target for many inflammation‐related diseases 6, 7, 8, 9, 10, 11, 12. Previous research has indicated that various molecules such as transforming growth factor β 13, prostaglandin E2 (PGE2) 13, 14, indoleamine 2,3‐dioxygenase (IDO) 15, 16, suppressor of cytokine signalling 1 17, programmed death‐ligand 1 (PD‐L1) 18 and inducible nitric oxide synthase (iNOS) 19, 20 secreted by MSCs mediate their immunosuppressive effects, whereas other studies have reported that cell‐to‐cell contact is the major mechanism of MSC‐mediated immunosuppression 21, 22. These discrepancies may be as a result of interspecies variation. For example, mouse and rabbit MSCs utilize iNOS, which leads to the suppression of STAT5 phosphorylation in T cells and the induction of immune cell apoptosis, whereas human and monkey MSCs use IDO, which leads to the degradation of tryptophan along the kynurenine pathway and the inhibition of T cell receptor‐induced proliferation 23, 24. Despite these findings, the detailed mechanisms of MSC‐mediated immunosuppression remain unclear.

A20, also known as tumour necrosis factor‐α‐induced protein 3 (TNFAIP3), is an ubiquitin‐modifying enzyme that has amino‐terminal deubiquitinating activity mediated by its ovarian tumour domain, and has a carboxy‐terminal zinc finger domain that supports E3 ubiquitin ligase activity. The up‐regulation of A20 can be induced by TNF‐α 25 and other proinflammatory cytokines *via* cytokine receptor engagement 26, which subsequently negatively regulates the NF‐κB 27, 28 or MAPK 29 pathways. Previous research has shown that expression of A20 by immune cells, such as dendritic cells (DCs) and macrophages, maintains immune homeostasis and prevents autoimmune diseases 30. Although A20 is believed to be an effective anti‐inflammatory and immunosuppressive protein in many cell types, little is known about the function of A20 in MSCs. As MSCs and A20 are both critical negative regulators of inflammation, we hypothesized that A20 plays a role in the immunoregulatory functions of MSCs, and this was investigated herein.

## Materials and methods

### Ethics statement

This study was conducted in strict accordance with national guidelines for the use of animals in scientific research, and was approved by the Animal Care and Use Committee of the Beijing Institute of Basic Medical Sciences (approval number BMS‐1104139).

### Mice

Male C57BL/6 mice (6–8 weeks old) were purchased from the Laboratory Animal Center, Academy of Military Medical Sciences, Beijing, China, and were maintained in a specific pathogen‐free mouse facility.

### Cell culture

Primary murine MSCs derived from murine bone marrow were isolated and cultured as we previously described 31. C3H/10T1/2, Clone 8 cells (ATCC, Manassas, VA, USA), a murine bone marrow‐derived mesenchymal cell line isolated from C57BL/6 mice, were cultured in minimal essential medium (MEM) with 2‐mM L‐glutamine, 1.5‐g/l sodium bicarbonate, 100‐U/ml penicillin, 100‐U/ml streptomycin and 10% foetal bovine serum (FBS). B16‐F0 cells (ATCC), a murine melanoma cell line isolated from C57BL/6, were cultured in DMEM supplemented with 10% FBS. All cells were cultured in a humidified atmosphere with 5% CO_2_ at 37°C.

### Lentiviral vector transduction

Lentivirus targeting mouse A20 (5′‐CAAAGCACUUAUUGACAGA‐3′) and the corresponding control virus were purchased from Genechem (Shanghai, China). 1 × 10^5^ C3H/10T1/2 cells were seeded in six‐well plates in serum‐ and antibiotic‐free MEM the day before transduction. After 24 hrs, C3H/10T1/2 cells were transduced with lentivirus expressing murine A20 shRNA (shA20 C3 MSCs) or control lentivirus (shCTRL C3 MSCs) in the presence of 10 μg/ml polybrene (Santa Cruz Biotechnology, Dallas, TX, USA) for 6 hrs. Transduced cells were selected with puromycin (Sigma‐Aldrich, St. Louis, MO, USA) at a concentration of 5 μg/ml for 48 hrs.

### Flow cytometric analysis

For surface molecule staining, cells were harvested with 0.25% trypsin and stained for 30 min. at 4°C. Antibodies against mouse CD45, CD105, CD44, IA/IE, CD11b, CD31, Sca‐1, CD29, intercellular cell adhesion molecule (ICAM), vascular cell adhesion molecule (VCAM) and PD‐L1 were purchased from BioLegend (San Diego, CA, USA). After washing three times in PBS, cells were fixed in 1% paraformaldehyde. Data were collected from 50,000 events for each sample with a BD FACSCalibur (BD Biosciences, San Jose, CA, USA), and date were analysed with FlowJo software version 7.6 (TreeStar, Ashland, OR, USA).

### Proliferation assay

Cell proliferation was measured with immunofluorescent staining of incorporated bromodeoxyuridine (BrdU) with a commercially available kit (BD Biosciences) according to the manufacturer's instructions. Briefly, cells were seeded at a density of 1 × 10^5^/well in six‐well plates, 10 μM BrdU was added and then the cells were incubated for 1.5–3 hrs before following the recommended staining protocol.

### Differentiation assay

To induce adipogenic differentiation, MSCs were cultured in DMEM supplemented with 10% FBS, 1‐μM dexamethasone, 200‐μM indomethacin, 0.5‐μM 3‐isobuty1‐1‐methyl‐xanthine and 10‐μg/ml insulin in 24‐well plates for 10 days. Osteogenic differentiation was induced in DMEM supplemented with 10% FBS, 0.1‐μM dexamethasone, 100‐μM ascorbate‐2 phosphate and 10‐mM β‐glycerophosphate in 24‐well plates for 2 weeks. Adipogenic and osteogenic induction were assayed with Oil Red O and alkaline phosphatase (ALP) staining, respectively as previously described 17. All reagents used in the MSC differentiation assay were purchased from Sigma‐Aldrich.

### Carboxy fluorescein diacetate succinimidyl ester labelling

Spleen cells were prepared as a single cell suspension, and dead cells were removed by density gradient centrifugation. CD3^+^ T cells were selected with a CD3ε MicroBead Kit (Miltenyi Biotec, Bergisch Gladbach, Germany), and then labelled with 5‐μM carboxy fluorescein diacetate succinimidyl ester (CFSE; Invitrogen, Carlsbad, CA, USA) for 7 min. at room temperature in the dark with gentle vortexing every 2 min. Cell labelling was terminated by adding 4–5 volumes of cold complete media. After washing, the spleen cells were stimulated with 50 ng/ml phorbol myristate acetate (PMA) and 1 μg/ml ionomycin (Sigma‐Aldrich), and co‐cultured with shCTRL MSCs or shA20 MSCs for 48 hrs. CD3^+^ T cell proliferation was measured by the reduction in CFSE fluorescence intensity *via* flow cytometry.

### Detection of cytokines and NO

Mesenchymal stem cells were stimulated with IFN‐γ and TNF‐α for 12 hrs or at indicated time points, and PGE2 and interleukin (IL)‐10 secretion were measured by ELISA (eBioscience, San Diego, CA, USA). Nitric oxide was detected with a modified Griess reagent (Sigma‐Aldrich). NO_3_ was converted into NO_2_ by nitrate reductase, and then NO_2_ production was measured by a modified Griess reagent kit (Sigma‐Aldrich).

### qRT‐PCR analysis

For qRT‐PCR analysis, total RNA was isolated from MSCs with TriPure (Roche, Indianapolis, IN, USA) then reverse transcribed into cDNA by a reverse transcriptase kit (Toyobo, Osaka, Japan) according to the manufacturer's protocol. qRT‐PCR was then performed with a SYBR Green PCR kit (Toyobo) on the Mx3005p system (Agilent Technologies, Santa Clara, CA, USA) to determine the expression levels of the genes of interest. Primer pairs were as follows: mouse β‐actin: forward 5′‐CTTCCGCCTTAATACTTC‐3′ and reverse 5′‐AAGCCTTCATACATCAAG‐3′; mouse A20: forward 5′‐ACCATGTTTGAAGGGTACTG‐3′ and reverse 5′‐GGCTACCTGTGTAGTTCGAG‐3′; mouse TNF‐α: forward 5′‐GATGGGTTGTACCTTGTCTACT‐3′ and reverse 5′‐CTTTCTCCTGGTATGAGATAGC‐3′; mouse IL‐10: forward 5′‐CCAAGCCTTATCGGAAATGA‐3′ and reverse 5′‐TCTCACCCAGGGAATTCAAA‐3′; mouse ALP: forward 5′‐GGGCAATGAGGTCACATCCA‐3′ and reverse 5′‐GTGGTTCACCCGAGTGGTAG‐3′; mouse Osterix: forward 5′‐ACTCATCCCTATGGCTCGTG‐3′ and reverse 5′‐GGTAGGGAGCTGGGTTAAGG‐3′; mouse peroxisome proliferator‐activated receptor‐γ (PPAR‐γ): forward 5′‐TTGATTTCTCCAGCATTTCT‐3′ and reverse 5′‐GCACTTTGGTATTCTTGGAG‐3′; mouse aP2: forward 5′‐AAGGTGAAGAGCATCATAACCCT‐3′ and reverse 5′‐TCACGCCTTTCATAACACATTCC‐3′. qRT‐PCR was performed with the following conditions: 95°C for 3 min.; 40 cycles: 95°C for 15 sec., 60°C for 15 sec., 72°C for 15 sec.; followed by melting curve analysis.

### Western blot analysis

Protein samples prepared in SDS sample buffer were boiled and separated on 12% SDS‐polyacrylamide gel, and then the proteins were transferred to a 0.45‐μm polyvinylidene fluoride blotting membranes. The membranes were then incubated with primary antibodies against the proteins of interest in blocking solution, washed and then incubated with HRP‐conjugated secondary antibodies. Finally, an enhanced chemiluminescence substrate (Thermo Fisher, Waltham, MA, USA) was added to the membranes prior to film exposure to detect the proteins according to manufacturer instructions.

### Analysis of *in vivo* tumour growth

B16‐F0 murine melanoma cells were expanded in DMEM supplemented with 10% FBS. Each mouse was subcutaneously injected into the right posterior flank with 5 × 10^5^ B16‐F0 cells in 100‐μL PBS, with or without co‐injection of shCTRL MSCs or shA20 MSCs (1 × 10^6^ cells/mouse), and PBS alone served as a control. Tumour growth was measured every 2 days, and tumour volume was calculated by the following formula: tumour volume = 0.5 × width^2^ × length. Mice were killed 13 days later when tumours began to hinder mobility, and the tumours were subsequently excised and weighed. Each experimental group included at least four mice.

### Statistical analysis

All data were analysed with Prism 5.0 software (GraphPad Software, CA, USA) and are presented as the mean ± S.D. Statistical significance was assessed by unpaired two‐tailed Student's *t*‐tests. *P* values less than 0.05 were considered significant.

## Results

### Inflammatory cytokines induce A20 expression in MSCs

**Figure 1:**
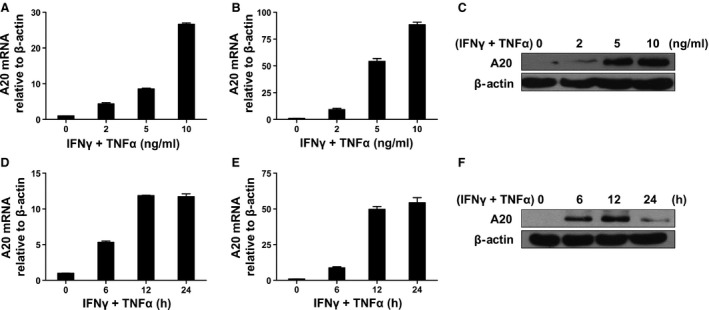
Inflammatory cytokines induce A20 expression in MSCs. MSCs (**A**) and C3H/10T1/2 (**B** and **C**) were treated with 0, 2, 5 and 10 ng/ml IFN‐γ and TNF‐α for 24 hrs. A20 mRNA and protein levels were examined by qRT‐PCR and Western blot analysis. MSCs (**D**) and C3H/10T1/2 (**E** and **F**) were treated with 5 ng/ml IFN‐γ and TNF‐α for 0, 6, 12 and 24 hrs, and mRNA and protein expression levels were determined by qRT‐PCR and Western blot analysis, respectively.

A20 transcription has been shown to be up‐regulated in various antigen‐presenting cells by numerous stimuli, such as TNF‐α, IFN‐γ and IL‐1β. To investigate the possible role of A20 in MSC‐mediated immunosuppression, we analysed the expression of A20 mRNA and protein following cytokine stimulation in MSCs from murine bone marrow and the C3H/10T1/2 murine MSC cell line, which has been utilized in several *in vitro* and *in vivo* studies 17, 32. Specifically, to assess the ability of inflammatory cytokines to induce A20 expression in MSCs, we added increasing concentrations of IFN‐γ and TNF‐α to MSCs for 24 hrs or 5 ng/ml IFN‐γ and TNF‐α for 6, 12 and 24 hrs. qRT‐PCR and Western blot analyses revealed that A20 expression was increased significantly in a dose‐dependent manner in both bone marrow–derived MSCs and C3H/10T1/2 (Fig. 1A–C). In addition, A20 expression was also highly responsive to stimulation with inflammatory cytokines at a set concentration of 5 ng/ml at the various time points (Fig. 1D–F).

Collectively, these results indicate that A20 mRNA and protein expression increase under inflammatory conditions in both murine bone marrow–derived MSCs and the C3H/10T1/2 murine MSC cell line. Considering that we observed similar responses between bone marrow MSCs and the MSC cell line to inflammatory factors, and that the latter is more readily available for *in vitro* and *in vivo* studies while reducing the number of animals required, all subsequent experiments were performed with the C3H/10T1/2 MSC cell line, which are hereafter referred to as C3 MSCs.

### A20 knockdown does not affect MSC morphology or phenotypic characteristics

**Figure 2:**
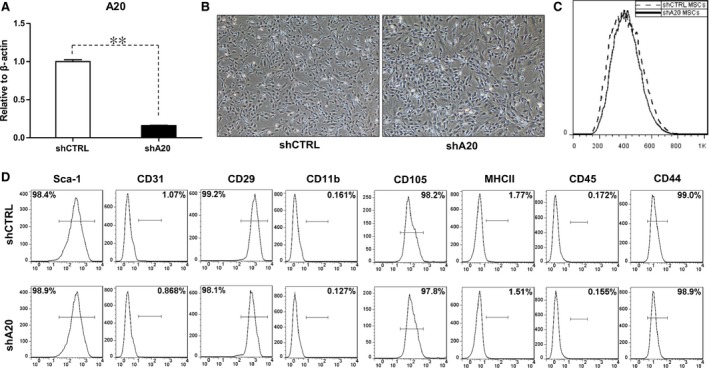
Morphological and phenotypic characterization of C3 MSCs after A20 knockdown. (**A**) qRT‐PCR analysis of A20 mRNA levels with or without A20 knockdown. (**B**) Morphology and (**C**) size of cultured shCTRL C3 MSCs and shA20 C3 MSCs were analysed by microscopy and flow cytometry. (**D**) Cell surface markers were analysed by flow cytometry, **P < 0.01. All experiments were repeated three times.

As both the qRT‐PCR and Western blot analyses revealed that there was a significant increase in A20 expression induced by inflammatory factors, we hypothesized that A20 is involved in regulating the immunosuppressive capacity of MSCs. Therefore, a lentivirus encoding A20 shRNA was used to silence A20 expression in mouse C3 MSCs, and the resulting effects were examined. Specifically, cells were transduced with a control lentivirus (shCTRL C3 MSCs) or lentivirus encoding for A20 shRNA (shA20 C3 MSCs), and the qRT‐PCR results showed a significant decrease in A20 expression after transduction with the lentivirus containing A20 shRNA (Fig. 2A).

We next assessed the morphological and phenotypic characteristics of shCTRL C3 MSCs and shA20 C3 MSCs. Both groups exhibited strong adherence and spindle‐shaped morphology (Fig. 2B). In addition, flow cytometry results showed that there was no significant difference in cell size between the experimental and control groups (Fig. 2C). The transduced MSCs were then analysed for cell surface molecule expression, and were found to express the MSC markers Sca‐1, CD105, CD29 and CD44, but were negative for the haematopoietic and endothelial markers CD45, CD11b, CD31 and MHC class II (Fig. 2D). These results indicate that C3 MSCs exhibited similar morphology and immunophenotype with or without A20 knockdown.

### Impaired adipogenic differentiation of MSCs with A20 knockdown

**Figure 3:**
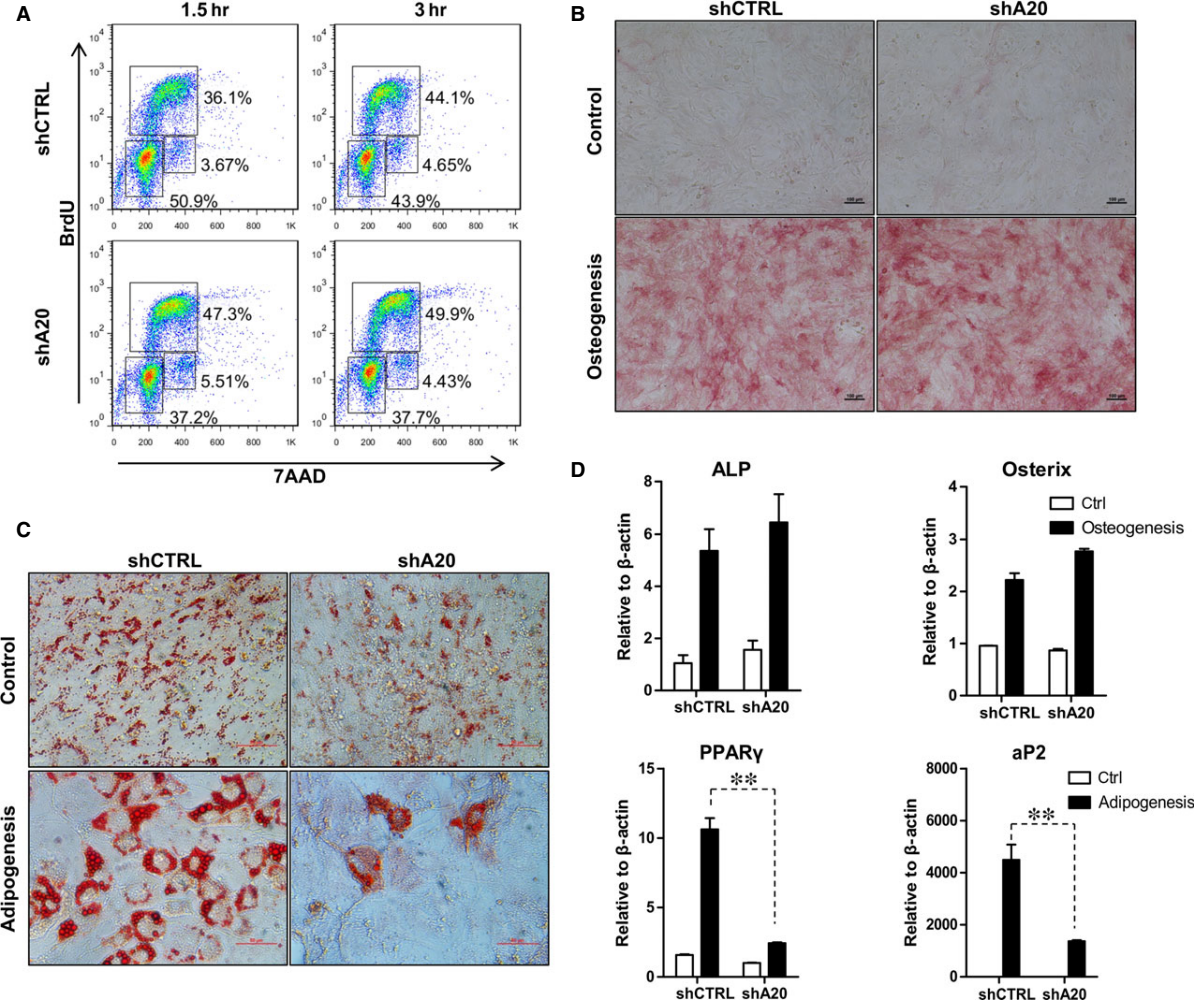
Proliferation and differentiation capacities of shCTRL C3 MSCs and shA20 C3 MSCs. (**A**) BrdU incorporation coupled with 7AAD staining was analysed by flow cytometry. C3 MSCs were cultured in (**B**) osteogenic or (**C**) adipogenic differentiation medium for 14 days, and stained with ALP (bar = 100 μm) or Oil Red O (bar = 50 μm). (**D**) C3 MSCs cultured in osteogenic or adipogenic medium for the indicated times were analysed for ALP, Osterix, PPAR‐γ and aP2 by qRT‐PCR, **P < 0.01.

Next, we analysed the effect of A20 knockdown on C3 MSC proliferation at different time points. In the proliferation assay, BrdU is incorporated into the DNA of proliferating cells and then detected with anti‐BrdU fluorescent antibodies, and 7AAD is coupled with BrdU staining to analyse the cell‐cycle phase by flow cytometry. Our results showed that there was a significantly higher proportion of shA20 C3 MSCs in S phase and a lower proportion in G0/G1 phase compared with those of shCTRL C3 MSCs (Fig. 3A). As previously reported, MSCs can differentiate into adipocytes and osteoblasts *in vitro* when cultured under appropriate conditions 17, 31. Our results showed that A20 knockdown did not influence osteogenic differentiation of either shCTRL C3 MSCs or shA20 C3 MSCs (Fig. 3B and D). However, compared with that of shCTRL C3 MSCs, adipogenic differentiation was dramatically reduced in shA20 C3 MSCs (Fig. 3C). This reduction in adipogenesis was further observed through analysis of PPAR‐γ and aP2 mRNA expression levels (Fig. 3D).

### A20 knockdown attenuates the immunosuppressive capacity of MSCs *in vitro* and *in vivo*

**Figure 4:**
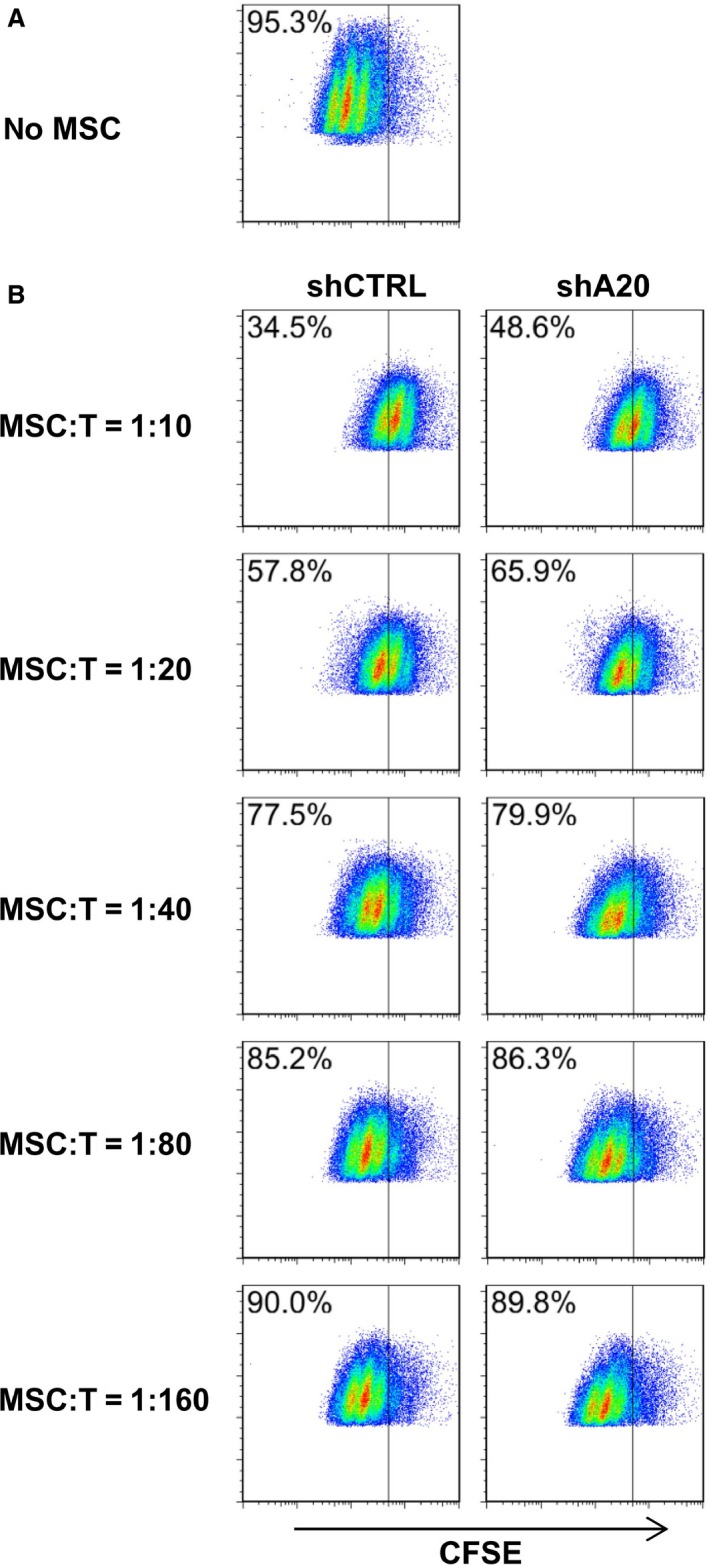
A20 knockdown attenuates immunosuppressive capacity of C3 MSCs *in vitro*. CFSE‐labelled CD3^+^ T cell was cultured alone (**A**) or co‐cultured with different numbers of C3 MSCs (**B**) in RPMI 1640 complete medium supplemented with PMA (50 ng/ml) and ionomycin (1 μg/ml) for 48 hrs. Cells were subjected to flow cytometry for T cell proliferation as detected by the CFSE signal.

**Figure 5:**
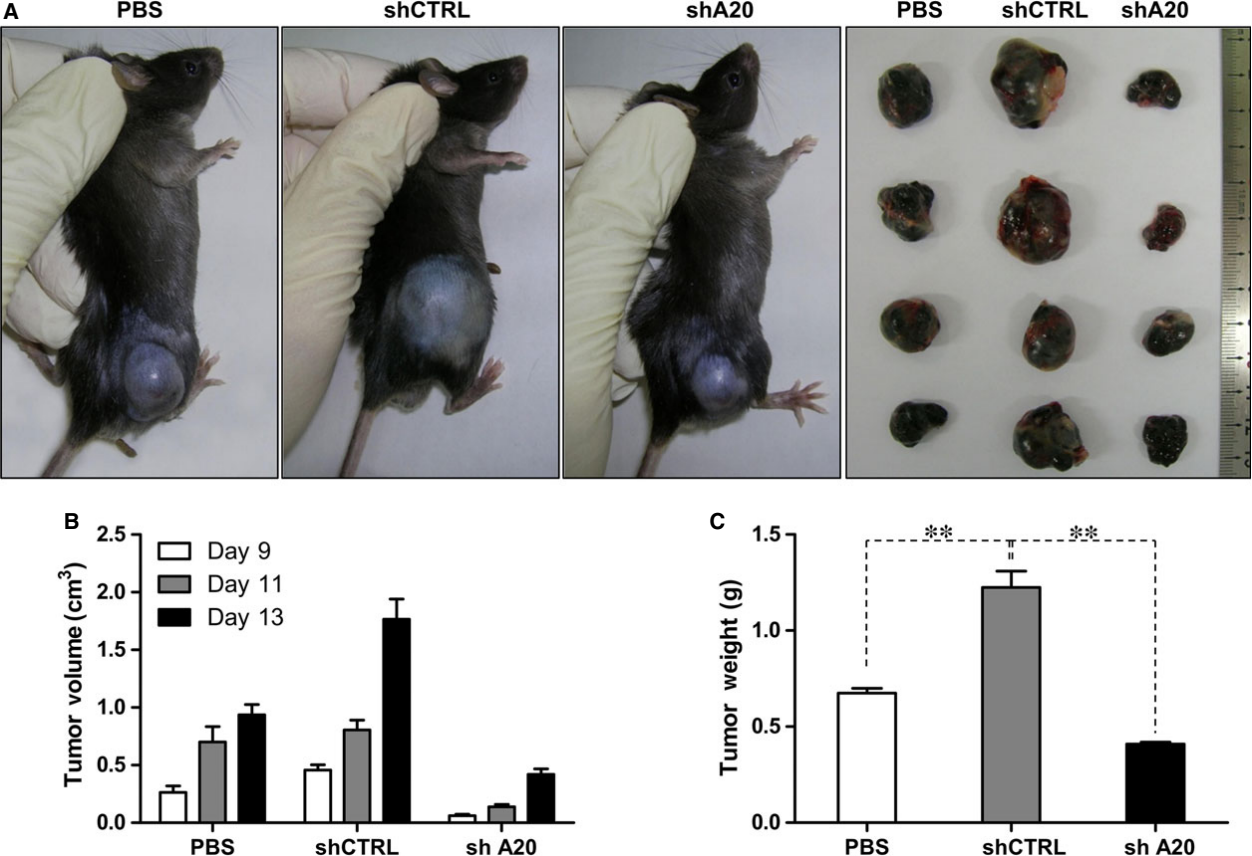
A20 knockdown inhibits tumorigenesis *in vivo*. (**A**) Representative tumours photographed 13 days after C57BL/6 mice were injected with PBS, shCTRL C3 MSCs or shA20 C3 MSCs. (**B**) On day 0, 5 × 10^5^ B16‐F0 cells in 100‐μl PBS were injected subcutaneously into the right posterior flanks, with or without co‐injection of shCTRL C3 MSCs or shA20 C3 MSCs (1 × 10^6^ cells). Tumour growth was measured at 2‐day intervals, and tumour volume was calculated. (**C**) Thirteen days later, all mice were killed and tumours were sectioned and weighted. Each treatment group included four mice, and data are representative of three independent experiments, **P < 0.01.

We next examined the immunosuppressive capacity of C3 MSCs following A20 knockdown *in vitro* and *in vivo*. Because A20 is a well‐known immunosuppressive molecule, we hypothesized that shA20 C3 MSCs would exhibit immune‐enhancing activity. To investigate this hypothesis, CD3^+^ T cells were labelled with CFSE, stimulated with PMA and ionomycin, and then co‐cultured with C3 MSCs. Changes in CFSE signals were then measured *via* flow cytometry to monitor T cell proliferation. We found that T cell proliferation was inhibited by shCTRL C3 MSCs as indicated by a slower reduction in CFSE fluorescence intensity. As expected, shA20 C3 MSCs partially reversed this immunosuppressive effect on T cell proliferation, as there was a faster reduction in CFSE fluorescence intensity for shA20 C3 MSCs than was observed for shCTRL C3 MSCs (Fig. 4). To further investigate this observation, we used an *in vivo* mouse melanoma tumour model. Specifically, B16‐F0 cells were subcutaneously injected into the right posterior flanks of C57BL/6 mice with co‐injections of shCTRL C3 MSCs, shA20 C3 MSCs or PBS. Thirteen days later, mice co‐injected with shCTRL C3 MSCs exhibited a 1.9‐fold greater tumour volume and 1.8‐fold greater tumour weight compared with those of mice co‐injected with PBS (Fig. 5A–C). However, in mice co‐injected with shA20 C3 MSCs, we observed only 0.45‐ and 0.61‐fold increases in tumour volume and weight, respectively, compared those of PBS‐co‐injected control mice (Fig. 5A–C). Overall, these data indicate that there is a role for A20 as an immunosuppressive attenuator of C3 MSCs, for when A20 was knocked down, the immunosuppressive effect of MSCs was attenuated.

### TNF‐α and IL‐10, but not ICAM, VCAM, PD‐L1, nitric oxide or PGE2 attenuate the immunosuppressive capacity of shA20 C3 MSCs

**Figure 6:**
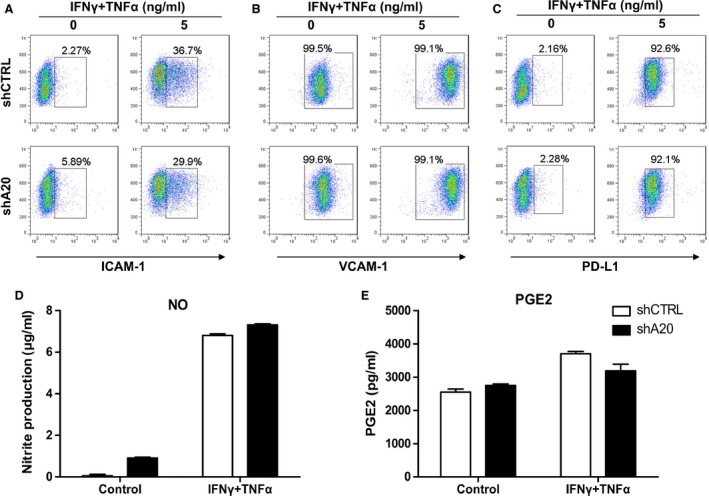
A20 knockdown showed no significant effect on ICAM‐1, VCAM‐1, PD‐L1, PGE2 or nitric oxide expression. ShCTRL C3 MSCs and shA20 C3 MSCs were cultured with or without stimulation with 5 ng/ml IFN‐γ and TNF‐α. 12 hrs later, the expression of (**A**) ICAM‐1, (**B**) VCAM‐1 and (**C**) PD‐L1 were analysed by flow cytometry. (**D**) Nitric oxide production was measured by Griess assay. (**E**) PGE2 production was measured by ELISA.

**Figure 7:**
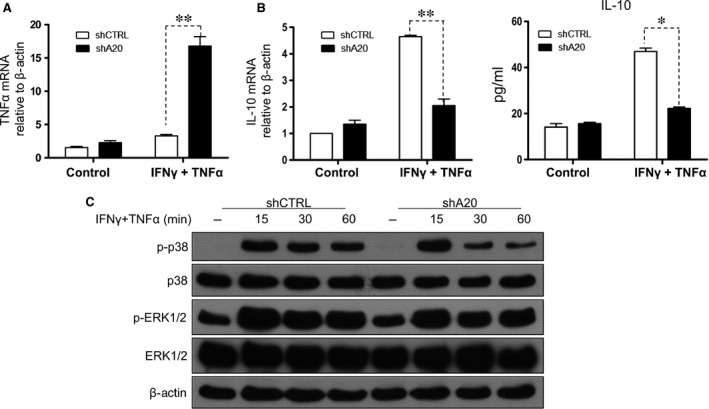
A20 inhibits TNF‐α and promotes IL‐10 production in C3 MSCs, and the p38/MAPK pathway is involved in A20‐induced immunomodulation. TNF‐α (**A**) and IL‐10 (**B**, left) expression was examined with or without stimulation with 5 ng/ml IFN‐γ and TNF‐α by qRT‐PCR. IL‐10 protein level was determined by ELISA (**B**, right). (**C**) The time courses of p38 phosphorylation in response to 5 ng/ml IFN‐γ and TNF‐α was determined by immunoblotting, **P < 0.01, *P < 0.05.

Many molecules such as ICAM‐1, VCAM‐1, PD‐L1, PGE2 and nitric oxide have been described to have major roles in MSC‐mediated immunosuppression. To investigate the mechanism by which A20 reverses the immunosuppressive capacity of MSCs, we treated shCTRL or shA20 C3 MSCs with IFN‐γ and TNF‐α at increasing concentrations or left them untreated. We then evaluated the expression of ICAM‐1, VCAM‐1 and PD‐L1 by flow cytometry, and the production of PGE2 and nitric oxide by ELISA and a Griess reaction, respectively. We found that ICAM‐1, PD‐L1, PGE2 and nitric oxide were up‐regulated in C3 MSCs stimulated with IFN‐γ and TNF‐α in a dose‐dependent manner, with no differences observed between shCTRL C3 MSCs and shA20 C3 MSCs (Fig. 6A and C–E). No significant changes in VCAM‐1 expression were observed with or without stimulation (Fig. 6B). We then compared the mRNA levels of representative inflammatory cytokines between shCTRL C3 MSCs and shA20 C3 MSCs by qRT‐PCR. Without stimulation, both shCTRLC3 MSCs and shA20 C3 MSCs exhibited low levels of TNF‐α and IL‐10 production (Fig. 7A and B). With stimulation, TNF‐α mRNA was induced in shCTRL C3 MSCs at a lower level than that in shA20 C3 MSCs (Fig. 7A). Conversely, IL‐10 mRNA and protein were induced in shCTRL C3 MSCs at a significantly higher level compared with that in shA20 C3 MSCs (Fig. 7B). Taken together, these results indicate the unique effects of A20 on the inhibition of TNF‐α and promotion of IL‐10 production in C3 MSCs.

### The MAPK pathway is involved in A20‐induced immunosuppression

Next, we investigated which pathway was involved in the regulation of TNF‐α and IL‐10 expression in MSCs. The MAPK pathway is one of the major intracellular signalling cascades and is activated by many stimuli, including IFN‐γ and TNF‐α. Therefore, we examined the MAPK pathway in C3 MSCs with and without A20 knockdown, and found that A20 knockdown inhibited the phosphorylation of p38 MAPK. Both shCTRL and shA20 C3 MSCs exhibited phosphorylation of p38 MAPK within 15 min. after IFN‐γ and TNF‐α stimulation, but surprisingly, p38 activation was rapidly inhibited within 30 min. in shA20 C3 MSCs (Fig. 7C). This finding reveals a possible role of A20 in the regulation of p38 MAPK signalling. Collectively, these data suggest that shA20 C3 MSCs have inhibited activation of the p38 MAPK pathway, which may lead to enhanced TNF‐α and decreased IL‐10 production.

## Discussion

Mesenchymal stem cells are present in nearly all tissues and can differentiate into various specialized cell types. The immunosuppressive ability of MSCs has been studied both *in vitro* and *in vivo* over the last few years, but the detailed mechanisms of their immunosuppression remain controversial. The results of this study reveal for the first time that A20 plays an important role in the immunomodulatory capacity of MSCs.

A20 is a ubiquitin‐modifying enzyme and is expressed by virtually all cell types, including macrophages, DCs and B cells, but has different functions in different cell types 33, 34. Here, we found that shA20 MSCs exhibit an enhanced proliferative capacity as has been demonstrated for other immune cells, which is possibly as a result of increased expression of NF‐κB‐dependent, anti‐apoptotic proteins. Dorronsoro *et al*. reported that A20 is a key regulator in human adipogenesis 35. In this study, we showed that the adipogenic differentiation capacity was dramatically decreased in shA20 C3 MSCs.

In spite of its function on many immune cells, such as DCs, T cells and B cells; however, whether A20 plays a role in MSC‐mediated immunosuppression remained unknown. Several molecules such as ICAM‐1, VCAM‐1, PD‐L1 and nitric oxide have been proposed in the mechanisms of immunosuppression by mouse MSCs, and although our results were in agreement with those of prior studies 18, 19, 36, these factors cannot explain the differences in immunomodulation between control and A20 knockdown MSCs observed herein. Specifically, the results of this study revealed that MSCs with A20 knockdown exert anti‐tumorigenic effects as documented in a melanoma tumour model. *In vitro*, we found that A20 attenuated the MSC‐mediated immunosuppressive effects on T cell proliferation by inhibiting IL‐10 and inducing TNF‐α. Interleukin‐10 is a key inhibitor of inflammatory responses that regulates the production of cytokines such as TNF‐α, and IL‐10 expression can be induced by p38 MPAK pathway activation in macrophages 37, 38, 39. Here, we further demonstrated that A20 knockdown inhibited the activation of p38 MAPK, promoted the production of TNF‐α, decreased the production of IL‐10 and partly reversed the immunosuppressive effects of MSCs.

In recent years, there have been many conflicting reports on the immunomodulatory effect of MSCs 40, 41, as MSCs have complex immune modulating activities. Thus, a new paradigm has been proposed for MSCs, in which MSC1 is classified as pro‐inflammatory and MSC2 as immunosuppressive 42, 43. When with cancer cells, MSC1‐treated groups showed higher levels of pro‐inflammatory factors, whereas MSC2‐treated groups had marked increases in anti‐inflammatory factors including IL‐10 21, 23, 42, 43. Considering that the detailed mechanisms of MSC‐mediated immunosuppression remain unclear, our results herein may offer possible guidance on the conflict in that MSC1 may have a lower expression of A20, which could reverse the immunosuppressive effect on T cell proliferation.

## Conclusions

This study revealed that A20 knockdown in MSCs reduced MSC‐mediated immunosuppression of T cell proliferation *in vitro* and reversed MSC‐mediated immunosuppression against tumour cells leading to decreased tumour growth *in vivo*. Our results further demonstrated the crucial role of A20 in MSC‐mediated expression of TNF‐α and IL‐10 in an inflammatory environment. Hence, this study provides new insights into the molecular mechanisms of immunoregulation by MSCs and provides a target for future clinical treatments.

## Conflict of interest

The authors declare that they have no competing interests.

## Author contribution

XXJ and NW conceived and designed the experiments. RJD, YMY, LZ, DCC, BX, PL, QL, YW, QYW and FX performed the experiments. RJD, YMY, LZ, DCC, BX, PL, QL, YW, QYW, FX, XXJ and NW analysed the data. NM, CW XXJ and NW contributed reagents, materials and analysis tools. RJD, YMY, LZ, XXJ and NW wrote this study. All authors read and approved the manuscript.
